# Artificial Teeth on Protruding Gums

**Published:** 1875-04

**Authors:** W. E. Driscoll

**Affiliations:** Bedford, Indiana


### ARTIFICIAL TEETH ON PROTRUDING GUMS.

BY W. E. DRISCOLL, BEDFORD, INDIANA.

All dentists, ("dentificers" or "dentificians" if Drs. Allport, Mills, *et al* prefer) have been annoyed with cases where the anterior part of the superior maxilla protrude so much that the most carefully adjusted porcelain gums or plain teeth can be easily detected as to their artificial character.

I need not remind the dentist (or "mechanician") how unsatisfactory such a result is to him or his patient. The remedy which I now suggest may have been tried by others, but they have failed to give their brethren the benefit of their experience so far as I know. And if I did not feel it a duty to give this to the profession I am sure I have no other motive in claiming attention. The method is much more practicable and satisfactory than may seem from the description I give which is as follows.

If the temporary set can be ready to place in the mouth within twenty-four hours after the teeth are extracted, proceed in all respects as usual until the model is ready for the reception of the teeth. Select plain teeth somewhat larger than usual for the six anterior ones, and let them *into* the model exactly where the natural ones stood, say at least one-fourth of an inch when the gum is very prominent. All the teeth, posterior to these six, to be set as usual and the plate finished up and placed in the mouth as soon as possible, when it will be found that the six front teeth go so far up into the gums that they do not become uncovered by any subsequent shrinkage of the gums or alveoli. And can not if properly selected and adjusted be detected at conversational distance from natural teeth, something that can not be truly affirmed of many sets of artificial teeth even in the most favorable mouths, and *never* in mouths where the gums protrude.

Sometimes I extract all except the six front teeth, and wait for the gums to shrink perfectly. Then take an impression, before extracting the front teeth; from the model cut off the plaster teeth, and in their places sink the plain, artificial teeth one-fourth of an inch beyond or deeper than the margin of the gum, then place the back teeth in position in the usual way, wax up and finish. When the patient comes for the plate extract the remaining six front teeth and place the plate at once into position. In this way but one plate is needed as the gums can not shrink from the teeth.

If one or more of the front teeth have been out until the alveoli have closed, then place the plain teeth as usual in such a case, and the remainder as above directed.

When a temporary set must be replaced by a permanent one, the front teeth can be set in the same depressions that were occupied by the temporary ones. I never extract for a partial set of front teeth now, until the plate is made and ready to be placed immediately in the mouth before any swelling or healing can take place. In this way the patient is relieved of the necessity of going an hour without the teeth. This however will be a great objection with those who would like to impose all the inconvenience and trouble possible upon those who elect to have artificial teeth instead of having their old shells filled. But like some other changes time is bringing, I do not know what is to be done about it.

If any one shonld hesitate to place the artificial teeth in the sockets of the natural ones from fear of subsequent irritation or retarded healing, I will say I have had no trouble of that kind in an experience with the plan of two years or a little over. If the front teeth are decayed even with the gum, then set the artificial teeth a little deeper in the model than if not so decayed, and use the pod beak root forceps in extracting and cut the process over each root uniform with its fellows.

